# Supplementary material for: Posttraumatic Osteomyelitis Risks Associated with NLRP3 Gene Polymorphisms in the Chinese Population
Source: J Pers Med. 2023 Jan 30;13(2):253. doi: 10.3390/jpm13020253 (PMC9959692; doi:10.3390/jpm13020253)
Supplement: Supplementary file 1 [file jpm-13-00253-s001.zip › jpm-2086683-supplementary.pdf]

**Table S1. PCR primers and extension primers of the 25 SNPs.**

| SNP | PCR primers | Extension primers |
|---|---|---|
| rs2043211 | F: 5'-TGAGACACAGCGTCCAATAGT-3'<br>F: 5'-TCCAAGAAGATGATGAGACAGAGG-3' | 5'-ctgactgactgactgactgactgactGACACTCAGGAACAGCACGGA-3' |
| rs10754558 | F: 5'-GTGGAGTGTCGGAGAAGAGA-3'<br>F: 5'-GCTAATTACATGAGGTCACCAAGA-3' | 5'-ctgactgactgactgactgactgactgCAATGACAGCATCGGGTGTTGTT-3' |
| rs12185396 | F: 5'-GGATGTGCCTGTGAGATTATCTT-3'<br>F: 5'-CACTTCCTGAACCAACAATGC-3' | 5'-ctgCCTGTCAGACCCTGAAACCCA-3' |
| rs8299 | F: 5'-AGAGACTGACCGTTGAGAAGG-3'<br>F: 5'-ATCTTTGCCGATGAAACTGTAGT-3' | 5'-ctgacCAATACAGTCATCAGTGGAGTC-3' |
| rs580253 | F: 5'-GAGCACAAGACCTCTGACAG-3'<br>F: 5'-AATATCTACCAACCAGCCATTCAA-3' | 5'-ctgactgaCTCTGAGCAAGTCCCAGATATA-3' |
| rs501192 | F: 5'-GAGCACAAGACCTCTGACAG-3'<br>F: 5'-AATATCTACCAACCAGCCATTCAA-3' | 5'-ctgactgactgCAGGCCTGCCGTGGTGGTGAGT-3' |
| rs4796793 | F: 5'-AGTCTCTTGTAACTACGCTATCC-3'<br>F: 5'-GCCTCTCCTATCTGCTATTCAT-3' | 5'-ctgactgactgactgactgactgGGAGTGTATAAGGTGTGTAAGT-3' |
| rs2293152 | F: 5'-CAACAACGGCAGCCTCTC-3'<br>F: 5'-TCCCCAACAAAACTTACATCACAA-3' | 5'-ctgactgactgactgactgactgactgacTCACAAAGGGCCTCTGGCTGC-3' |
| rs7525979 | F: 5'-CCATCGGCAAGACCAAGAC-3'<br>F: 5'-CCAGGCTCCTCTGTGTCA-3' | 5'-ctgactgactgactgactgactgactgactgactgactgactgactgTTCTGAGCCTGTGCACA<br>C-3' |
| rs35829419 | F: 5'-CTGTCATCGGGTGGAGTCA-3' | 5'-ctgactgactgactgactgAGAGGAGCTTGGGAGGACACACT-3' |

| | | |
|---|---|---|
| rs1785929 | F: 5’-GCGAGGAAGCAGGAGGAA-3’<br>F: 5’-TTCAGCATCATGGCAGGAAT-3’<br>F: 5’-ACACATATCCCTTACTCCAGTTAA-3’ | 5’-ctgactgaAGGAGTTCAATGTTCAATGGAA-3’ |
| rs1785928 | F: 5’-TTCTAGCCAAACTCCTGACTCTT-3’<br>F: 5’-CAGTGAACATCCTTGCAGTACC-3’ | 5’-ctgactgactgactgacAGATGGTGTCCTACAGCAGCCA-3’ |
| rs1026916 | F: 5’-AACTGGAGTCTAGAACTGAATGA-3’<br>F: 5’-CCACGGACGTTTACCCTAA-3’ | 5’-cAAGTTTAACCCAAAGACTGT-3’ |
| rs744166 | F: 5’-GAGTACAAACCCTGAACCCTTT-3’<br>F: 5’-GGAGTGCCAACATTGAGAGG-3’ | 5’-ctgaATGTCTTGAGGGAATCGAGC-3’ |
| rs696 | F: 5’-GTACTTATATCCACACTGCACACT-3’<br>F: 5’-CAATGATCTTTCTCGTCCCCTAC-3’ | 5’-ctgactgATCCACACTGCACACTGCCT-3’ |
| rs4648068 | F: 5’-GGCTCATTGAACGTCTCTGT-3’<br>F: 5’-TTTAAGGGAACGACCATTATCTGT-3’ | 5’-ctgactgacTCTTGGTGAAATAATCCTTCA-3’ |
| rs530537 | F: 5’-TGCCAATCCCAACCACATC-3’<br>F: 5’-ATGAAGCCTTTAACTGAGACTTGT-3’ | 5’-ctgactgactCTTAATTTCACAAAACATTTGCA-3’ |
| rs681757 | F: 5’-AATCACAAGTAGGTCAACCCAAAT-3’<br>F: 5’-GGATACAATCTCACCTGAGTTCG-3’ | 5’-ctgactgactgactgaGTCAAGTCTTCCTCAGTTATCCA-3’ |
| rs559289 | F: 5’-TGGGACACTGGTGGGAAG-3’<br>F: 5’-AAGAAGATAGGGAGTTTGGCAAA-3’ | 5’-ctgactgactgactgactgacTCATTTGAAAGGTACTTGGAAC-3’ |
| rs556205 | F: 5’-TGTGCTTATTTCCTTATCTACTCC-3’<br>F: 5’-ACAAGAGGTATCTGAGGTCAC-3’ | 5’-ctgactgactgactgactgactgactgCCATGTAGACTGAGTATTGC-3’ |

| | | |
|---|---|---|
| rs2569190 | F: 5’-CCTCCTCTGTGAACCCTGAT-3’<br>F: 5’-GCTTAGGCTCCCGAGTCA-3’ | 5’-ctgaGCAGAATCCTTCCTGTTACGG-3’ |
| rs4612666 | F: 5’-GGTTGCACAACAATGTGAAGT-3’<br>F: 5’-ATAACAAGTAAGCATTCTCCAAGC-3’ | 5’-TTCTCCAAGCTCCCACCAATACTAC-3’ |
| rs1789547 | F: 5’-GAAGAAGAGATAAATGGGTTGTGGTAT-3’<br>F: 5’-TGGAATTATAGATATTACAGCTTGCCTTT-3’ | 5’-ctgactgactAGCTCTAGTTATTTGTTTTTATTGCA-3’ |
| rs2032206 | F: 5’-TGGGACACTGGTGGGAAG-3’<br>F: 5’-AAGAAGATAGGGAGTTTGGCAAA-3’ | 5’-ctgactgactgactgacTTGGTCATTATGGTGACTTAGG-3’ |
| rs1053004 | F: 5’-GGTTTTGAGTTGCCAAATCCG-3’<br>F: 5’-TCGTATGAGGGTGTATACCTG-3’ | 5’-CTGACTGATGTTCCCATACGCACAGGAGAGGC-3’ |

SNP: Single Nucleotide Polymorphism; PCR: Polymerase Chain Reaction; F: Forward; R: Reverse.

**Table S2. HWE test outcomes of the 25 gene polymorphisms for healthy controls.**

| **SNP** | **$\chi^2$ value** | ***P* value** |
|---|---|---|
| rs2043211 | 0.095 | 0.758 |
| rs10754558 | 0.779 | 0.377 |
| rs12185396 | 0.647 | 0.421 |
| rs8299 | 0.647 | 0.421 |
| rs580253 | **0.003** | 0.958 |
| rs501192 | **0.003** | 0.958 |
| rs4796793 | **0.008** | 0.927 |
| rs2293152 | 0.686 | 0.407 |
| rs7525979 | 0.752 | 0.386 |
| rs35829419 | **0.001** | 0.979 |
| rs1785929 | 1.253 | 0.263 |
| rs1785928 | 1.253 | 0.263 |
| rs1026916 | 0.034 | 0.853 |
| rs744166 | **0.004** | 0.950 |
| rs696 | 3.122 | 0.077 |
| rs4648068 | **0.002** | 0.967 |
| rs530537 | 0.304 | 0.582 |
| rs681757 | 0.511 | 0.475 |
| rs559289 | 0.511 | 0.475 |
| rs556205 | **0.003** | 0.958 |

| | | |
|---|---|---|
| rs2569190 | 1.057 | 0.304 |
| rs4612666 | 0.265 | 0.607 |
| rs1789547 | 0.151 | 0.697 |
| rs2032206 | 1.490 | 0.222 |

IL: Interleukin; HWE: Hardy-Weinber Equilibrium

**Table S3. Comparisons of genotype distribution, allele frequency, and genetic models of the remaining 21 SNPs between PTOM patients and healthy controls.**

| SNP | Item | Allele or genotype | Patients | Controls | *P* values | OR (95% CI) |
|---|---|---|---|---|---|---|
| rs2043211 | Genotype (n, %) | TT | 85 (27.78) | 108 (29.35) | 0.731 | NA |
| | | AT | 159 (51.96) | 180 (48.91) | | |
| | | AA | 62 (20.26) | 80 (21.74) | | |
| | Allele frequency | T vs. A | 329/283 | 396/340 | 0.987 | 0.998 (0.805, 1.238) |
| | Dominant model | TT+AT vs. AA | 244/62 | 288/80 | 0.640 | 1.093 (0.753, 1.587) |
| | Recessive model | TT vs. AT+AA | 85/221 | 108/260 | 0.654 | 0.926 (0.662, 1.296) |
| | Homozygous model | TT vs. AA | 85/62 | 108/80 | 0.945 | 1.016 (0.656, 1.572) |
| | Heterzygous model | AT vs. AA | 159/62 | 180/80 | 0.515 | 1.140 (0.768, 1.691) |
| rs2569190 | Genotype (n, %) | GG | 57 (18.63) | 58 (15.76) | 0.521 | NA |
| | | AG | 145 (47.39) | 188 (51.09) | | |
| | | AA | 104 (33.99) | 122 (33.15) | | |
| | Allele frequency | G vs. A | 259/353 | 304/432 | 0.707 | 1.043 (0.839, 1.296) |
| | Dominant model | GG+AG vs. AA | 202/104 | 246/122 | 0.819 | 0.963 (0.699, 1.328) |
| | Recessive model | GG vs. AG+AA | 57/249 | 58/310 | 0.325 | 1.224 (0.819, 1.829) |
| | Homozygous model | GG vs. AA | 57/104 | 58/122 | 0.535 | 1.153 (0.735, 1.807) |
| | Heterzygous model | AG vs. AA | 145/104 | 188/122 | 0.564 | 0.905 (0.644, 1.271) |
| rs1785929 | Genotype (n, %) | AA | 9 (2.94) | 9 (2.45) | 0.444 | NA |
| | | AG | 83 (27.12) | 116 (31.52) | | |
| | | GG | 214 (69.93) | 243 (66.03) | | |
| | Allele frequency | A vs. G | 101/511 | 134/602 | 0.412 | 0.888 (0.668, 1.179) |

| | | | | | | |
|---|---|---|---|---|---|---|
| | Dominant model | AA+AG vs. GG | 92/214 | 125/243 | 0.280 | 0.836 (0.603, 1.158) |
| | Recessive model | AA vs. AG+GG | 9/297 | 9/359 | 0.691 | 1.209 (0.474, 3.084) |
| | Homozygous model | AA vs. GG | 9/214 | 9/243 | 0.791 | 1.136 (0.443, 2.913) |
| | Heterzygous model | AG vs. GG | 83/214 | 116/243 | 0.226 | 0.812 (0.580, 1.137) |
| rs1789547 | Genotype (n, %) | TT | 7 (2.29) | 8 (2.17) | 0.830 | NA |
| | | CT | 76 (24.84) | 99 (26.90) | | |
| | | CC | 223 (72.88) | 261 (70.92) | | |
| | Allele frequency | T vs. C | 90/522 | 115/621 | 0.640 | 0.931 (0,690, 1.256) |
| | Dominant model | TT+CT vs. CC | 83/223 | 107/261 | 0.575 | 0.908 (0.648, 1.273) |
| | Recessive model | TT vs. CT+CC | 7/299 | 8/360 | 0.921 | 1.054 (0.378, 2.939) |
| | Homozygous model | TT vs. CC | 7/223 | 8/261 | 0.964 | 1.024 (0.366, 2.869) |
| | Heterzygous model | CT vs. CC | 76/223 | 99/261 | 0.547 | 0.898 (0.634, 1.273) |
| rs1785928 | Genotype (n, %) | AA | 9 (2.94) | 9 (2.45) | 0.444 | NA |
| | | AG | 83 (27.12) | 116 (31.52) | | |
| | | GG | 214 (69.93) | 243 (66.03) | | |
| | Allele frequency | A vs. G | 101/511 | 134/602 | 0.412 | 0.888 (0.668, 1.179) |
| | Dominant model | AA+AG vs. GG | 92/214 | 125/243 | 0.280 | 0.836 (0.603, 1.158) |
| | Recessive model | AA vs. AG+GG | 9/297 | 9/359 | 0.691 | 1.209 (0.474, 3.084) |
| | Homozygous model | AA vs. GG | 9/214 | 9/243 | 0.791 | 1.136 (0.443, 2.913) |
| | Heterzygous model | AG vs. GG | 83/214 | 116/243 | 0,226 | 0.812 (0.580, 1.137) |
| rs12185396 | Genotype (n, %) | CC | 212 (69.28) | 246 (66.85) | 0.761 | NA |
| | | CT | 85 (27.78) | 110 (29.89) | | |
| | | TT | 9 (2.94) | 9 (2.45) | | |

| | | | | | | |
|---|---|---|---|---|---|---|
| | Allele frequency | C vs. T | 509/103 | 602/128 | 0.734 | 1.051 (0.790, 1.397) |
| | Dominant model | CC+CT vs. TT | 297/9 | 356/9 | 0.704 | 0.834 (0.327, 2.129) |
| | Recessive model | CC vs. CT+TT | 212/94 | 246/119 | 0.602 | 1.091 (0.787, 1.531) |
| | Homozygous model | CC vs. TT | 212/9 | 246/9 | 0.757 | 0.862 (0.336, 2.211) |
| | Heterzygous model | CT vs. TT | 85/9 | 110/9 | 0.600 | 0.773 (0.294, 2.031) |
| rs681757 | Genotype (n, %) | GG | 9 (2.94) | 9 (2.45) | 0.634 | NA |
| | | GT | 81 (26.47) | 109 (29.62) | | |
| | | TT | 216 (70.59) | 250 (67.93) | | |
| | Allele frequency | G vs. T | 99/513 | 127/609 | 0.598 | 0.925 (0.694, 1.234) |
| | Dominant model | GG+GT vs. TT | 90/216 | 118/250 | 0.458 | 0.883 (0.635, 1.227) |
| | Recessive model | GG vs. GT+TT | 9/297 | 9/359 | 0.691 | 1.209 (0.474, 3.084) |
| | Homozygous model | GG vs. TT | 9/216 | 9/250 | 0.761 | 1.157 (0.451, 2.968) |
| | Heterzygous model | GT vs. TT | 81/216 | 109/250 | 0.385 | 0.860 (0.612, 1.209) |
| rs8299 | Genotype (n, %) | TT | 9 (2.94) | 9 (2.45) | 0.761 | NA |
| | | CT | 85 (27.78) | 110 (29.89) | | |
| | | CC | 212 (69.28) | 246 (66.85) | | |
| | Allele frequency | T vs. C | 103/509 | 128/602 | 0.734 | 0.952 (0.716, 1.266) |
| | Dominant model | TT+CT vs. CC | 94/212 | 119/246 | 0.602 | 0.917 (0.661, 1.271) |
| | Recessive model | TT vs. CT+CC | 9/297 | 9/356 | 0.704 | 1.199 (0.470, 3.058) |
| | Homozygous model | TT vs. CC | 9/212 | 9/246 | 0.757 | 1.160 (0.452, 2.977) |
| | Heterzygous model | CT vs. CC | 85/212 | 110/246 | 0.526 | 0.897 (0.640, 1.257) |
| rs2032206 | Genotype (n, %) | GG | 150 (49.02) | 181 (49.18) | 0.636 | NA |
| | | AG | 129 (42.16) | 147 (39.95) | | |

| | | | | | | |
|---|---|---|---|---|---|---|
| | | AA | 27 (8.82) | 40 (10.87) | | |
| | Allele frequency | G vs. A | 429/183 | 509/227 | 0.709 | 1.045 (0.828, 1.320) |
| | Dominant model | GG+AG vs. AA | 279/27 | 328/40 | 0.377 | 1.260 (0.754, 2.106) |
| | Recessive model | GG vs. AG+AA | 150/156 | 181/187 | 0.966 | 0.993 (0.734, 1.345) |
| | Homozygous model | GG vs. AA | 150/27 | 181/40 | 0.451 | 1.228 (0.720, 2.094) |
| | Heterzygous model | AG vs. AA | 129/27 | 147/40 | 0.342 | 1.300 (0.756, 2.236) |
| rs559289 | Genotype (n, %) | CC | 9 (2.94) | 9 (2.45) | 0.634 | NA |
| | | AC | 81 (26.47) | 109 (29.62) | | |
| | | AA | 216 (70.59) | 250 (67.93) | | |
| | Allele frequency | C vs. A | 99/513 | 127/609 | 0.598 | 0.925 (0.694, 1.234) |
| | Dominant model | CC+AC vs. AA | 90/216 | 118/250 | 0.458 | 0.883 (0.635, 1.227) |
| | Recessive model | CC vs. AC+AA | 9/297 | 9/359 | 0.691 | 1.209 (0.474, 3.084) |
| | Homozygous model | CC vs. AA | 9/216 | 9/250 | 0.761 | 1.157 (0.451, 2.968) |
| | Heterzygous model | AC vs. AA | 81/216 | 109/250 | 0.385 | 0.860 (0.612, 1.209) |
| rs4796793 | Genotype (n, %) | CC | 113 (36.93) | 134 (36.41) | 0.405 | NA |
| | | CG | 135 (44.12) | 175 (47.55) | | |
| | | GG | 58 (18.95) | 56 (15.22) | | |
| | Allele frequency | C vs. G | 361/251 | 443/287 | 0.527 | 0.932 (0.748, 1.160) |
| | Dominant model | CC+CG vs. GG | 248/58 | 309/56 | 0.215 | 0.775 (0.518, 1.160) |
| | Recessive model | CC vs. CG+GG | 113/193 | 134/231 | 0.954 | 1.009 (0.737, 1.383) |
| | Homozygous model | CC vs. GG | 113/58 | 134/56 | 0.364 | 0.814 (0.522, 1.270) |
| | Heterzygous model | CG vs. GG | 135/58 | 175/56 | 0.179 | 0.745 (0.484, 1.145) |
| rs744166 | Genotype (n, %) | GG | 63 (20.59) | 66 (17.93) | 0.680 | NA |

| | | | | | | |
|---|---|---|---|---|---|---|
| | | AG | 143 (46.73) | 179 (48.37) | | |
| | | AA | 100 (32.68) | 123 (33.42) | | |
| | Allele frequency | G vs. A | 269/343 | 311/425 | 0.531 | 1.072 (0.863, 1.331) |
| | Dominant model | GG+AG vs. AA | 206/100 | 245/123 | 0.838 | 1.034 (0.749, 1.428) |
| | Recessive model | GG vs. AG+AA | 63/243 | 66/302 | 0.383 | 1.186 (0.808, 1.742) |
| | Homozygous model | GG vs. AA | 63/100 | 66/123 | 0.469 | 1.174 (0.760, 1.813) |
| | Heterzygous model | AG vs. AA | 143/100 | 179/123 | 0.920 | 0.983 (0.697, 1.385) |
| rs1026916 | Genotype (n, %) | GG | 100 (32.68) | 123 (33.42) | 0.736 | NA |
| | | AG | 143 (46.73) | 178 (48.37) | | |
| | | AA | 63 (20.59) | 67 (18.21) | | |
| | Allele frequency | G vs. A | 343/269 | 424/312 | 0.564 | 0.938 (0.756, 1.165) |
| | Dominant model | GG+AG vs. AA | 243/63 | 301/67 | 0.435 | 0.859 (0.585, 1.260) |
| | Recessive model | GG vs. AG+AA | 100/206 | 123/245 | 0.838 | 0.967 (0.700, 1.335) |
| | Homozygous model | GG vs. AA | 100/63 | 123/67 | 0.511 | 0.865 (0.560, 1.334) |
| | Heterzygous model | AG vs. AA | 143/63 | 178/67 | 0.450 | 0.854 (0.568, 1.285) |
| rs2293152 | Genotype (n, %) | CC | 72 (23.53) | 96 (26.09) | 0.671 | NA |
| | | CG | 162 (52.94) | 190 (51.63) | | |
| | | GG | 72 (23.53) | 79 (21.47) | | |
| | Allele frequency | C vs. G | 306/306 | 382/348 | 0.395 | 0.911 (0.735, 1,129) |
| | Dominant model | CC+CG vs. GG | 234/72 | 286/79 | 0.560 | 0.898 (0.624, 1.291) |
| | Recessive model | CC vs. CG+GG | 72/234 | 96/269 | 0.409 | 0.862 (0.606, 1.226) |
| | Homozygous model | CC vs. GG | 72/72 | 96/79 | 0.387 | 0.823 (0.529, 1.280) |
| | Heterzygous model | CG vs. GG | 162/72 | 190/79 | 0.732 | 0.936 (0.639, 1.371) |

| | | | | | | |
|---|---|---|---|---|---|---|
| rs696 | Genotype (n, %) | TT | 48 (15.69) | 71 (19.29) | 0.224 | NA |
| | | CT | 154 (41.85) | 162 (44.02) | | |
| | | CC | 104 (33.99) | 135 (36.68) | | |
| | Allele frequency | T vs. C | 250/362 | 304/432 | 0.866 | 0.981 (0.789, 1.220) |
| | Dominant model | TT+CT vs. CC | 202/104 | 233/135 | 0.466 | 1.125 (0.819, 1.546) |
| | Recessive model | TT vs. CT+CC | 48/258 | 71/297 | 0.221 | 0.778 (0.520, 1.164) |
| | Homozygous model | TT vs. CC | 48/104 | 71/135 | 0.567 | 0.878 (0.561, 1.372) |
| | Heterzygous model | CT vs. CC | 154/104 | 162/135 | 0.222 | 1.234 (0.880, 1.730) |
| rs4648068 | Genotype (n, %) | GG | 61 (19.93) | 82 (22.28) | 0.224 | NA |
| | | AG | 160 (52.29) | 183 (49.73) | | |
| | | AA | 85 (27.78) | 103 (27.99) | | |
| | Allele frequency | G vs. A | 282/330 | 347/389 | 0.696 | 0.968 (0.773, 1.188) |
| | Dominant model | GG+AG vs. AA | 221/85 | 265/103 | 0.951 | 1.011 (0.721, 1.417) |
| | Recessive model | GG vs. AG+AA | 61/245 | 82/286 | 0.458 | 0.868 (0.598, 1.261) |
| | Homozygous model | GG vs. AA | 61/90 | 82/103 | 0.469 | 0.851 (0.551, 1.316) |
| | Heterzygous model | AG vs. AA | 160/85 | 183/103 | 0.751 | 1.059 (0.741, 1.514) |
| rs501192 | Genotype (n, %) | TT | 0 (0.0) | 0 (0.0) | NA | NA |
| | | CT | 3 (0.98) | 2 (0.55) | | |
| | | CC | 303 (99.02) | 363 (99.45) | | |
| | Allele frequency | T vs. C | 3/609 | 2/728 | 0.843 | 1.793 (0.299, 10.766) |
| | Dominant model | TT+CT vs. CC | 3/303 | 2/363 | 0.836 | 1.812 (0.301, 10.914) |
| | Recessive model | TT vs. CT+CC | 0/306 | 0/365 | NA | NA |
| | Homozygous model | TT vs. CC | 0/306 | 0/363 | NA | NA |

| | | | | | | |
|---|---|---|---|---|---|---|
| | Heterzygous model | CT vs. CC | 3/303 | 2/363 | 0.836 | 1.812 (0.301, 10.914) |
| rs580253 | Genotype (n, %) | AA | 0 (0.0) | 0 (0.0) | NA | NA |
| | | AG | 3 (0.98) | 2 (0.55) | | |
| | | GG | 303 (99.02) | 363 (99.45) | | |
| | Allele frequency | A vs. G | 3/609 | 2/728 | 0.843 | 1.793 (0.299, 10.766) |
| | Dominant model | AA+AG vs. GG | 3/303 | 2/363 | 0.836 | 1.812 (0.301, 10.914) |
| | Recessive model | AA vs. AG+GG | 0/306 | 0/365 | NA | NA |
| | Homozygous model | AA vs. GG | 0/303 | 0/363 | NA | NA |
| | Heterzygous model | AG vs. GG | 3/303 | 2/363 | 0.836 | 1.812 (0.301, 10.914) |
| rs556205 | Genotype (n, %) | GG | 0 (0.0) | 0 (0.0) | NA | NA |
| | | GT | 3 (0.98) | 2 (0.54) | | |
| | | TT | 303 (99.02) | 366 (99.46) | | |
| | Allele frequency | G vs. T | 3/609 | 2/734 | 0.836 | 1.808 (0.301, 10.854) |
| | Dominant model | GG+GT vs. TT | 3/303 | 2/366 | 0.836 | 1.812 (0.301, 10.914) |
| | Recessive model | GG vs. GT+TT | 0/306 | 0/368 | NA | NA |
| | Homozygous model | GG vs. TT | 0/303 | 0/366 | NA | NA |
| | Heterzygous model | GT vs. TT | 3/303 | 2/366 | 0.836 | 1.812 (0.301, 10.914) |
| rs530537 | Genotype (n, %) | CC | 10 (3.27) | 22 (5.98) | 0.193 | NA |
| | | CT | 132 (43.14) | 144 (39.13) | | |
| | | TT | 164 (53.59) | 202 (54.89) | | |
| | Allele frequency | C vs. T | 152/460 | 188/548 | 0.766 | 0.963 (0.752, 1.233) |
| | Dominant model | CC+CT vs. TT | 142/164 | 166/202 | 0.737 | 1.054 (0.777, 1.428) |
| | Recessive model | CC vs. CT+TT | 10/296 | 22/346 | 0.099 | 0.531 (0.248, 1.140) |

| | | | | | | |
|---|---|---|---|---|---|---|
| | Homozygous model | CC vs. TT | 10/164 | 22/202 | 0.138 | 0.560 (0.258, 1.216) |
| | Heterzygous model | CT vs. TT | 132/164 | 144/202 | 0.448 | 1.129 (0.825, 1.545) |
| rs1053004 | Genotype (n, %) | AA | 125 (40.98) | 143 (39.61) | 0.396 | NA |
| | | AG | 140 (45.90) | 157 (43.49) | | |
| | | GG | 40 (13.11) | 61 (16.90) | | |
| | Allele frequency | A vs. G | 390/220 | 443/279 | 0.333 | 1.116 (0.893, 1.395) |
| | Dominant model | AA+AG vs. GG | 265/40 | 300/61 | 0.175 | 1.347 (0.875, 2.074) |
| | Recessive model | AA vs. AG+GG | 125/180 | 143/218 | 0.719 | 1.059 (0.776, 1.444) |
| | Homozygous model | AA vs. GG | 125/40 | 143/61 | 0.225 | 1.333 (0.837, 2.123) |
| | Heterzygous model | AG vs. GG | 140/40 | 157/61 | 0.189 | 1.360 (0.859, 2.153) |

PTOM: Posttraumatic osteomyelitis; OR: odds ratio, CI: confidence interval; NA: not available.
